# Laparoscopic transcystic exploration of the common bile duct using a 9 Fr catheter

**DOI:** 10.3389/fsurg.2026.1566116

**Published:** 2026-02-26

**Authors:** Po Li, Jianrong Cheng, Yiru Hou, Feifei Cui, Xi Chen, Huiling Sun, Ruirui Ma, Jiaxi Yao, Xiaojun Chen

**Affiliations:** 1 Department of General Surgery, Zhangye Second People’s Hospital, Gansu, China; 2 Department of General Surgery, Lanzhou First People’s Hospital, Gansu, China; 3 Department of Medical School, Hexi University School of Medicine, Gansu, China; 4 Department of Urology, Hexi University Affiliated Zhangye People’s Hospital, Gansu, China; 5 Institute of Urology, Hexi University, Zhangye, Gansu, China

**Keywords:** biliary pancreatitis, choledochoscopy, common bile duct microstones, efficacy, endoscopy

## Abstract

**Objective:**

This study aimed to explore the clinical efficacy of performing a laparoscopy with a 9Fr disposable pancreaticobiliary catheter in diagnosing and treating common bile duct stones.

**Methods:**

Duodenoscopy was performed in 66 patients between January 2020 and January 2024 at our institution. Clinical indicators were analyzed, and postoperative treatment effects were monitored.

**Results:**

A total of 66 patients underwent surgery, including 50 with secondary common bile duct stones and 16 with primary common bile duct stones. The operative time ranged from 80 to 290 min (138.79 ± 36.86 min). Intraoperatively, blood loss volume ranged from 5 to 50 mL (13.03 ± 7.06 mL). The average postoperative length of hospital stay was 9.95 ± 2.43 days. The success rate of stone removal was 98.5%. One patient had the stone enter the pancreatic duct during the removal process, resulting in failed stone removal. Postoperative complications occurred in 7 patients. Three patients had fever and improved after antibiotic treatment. One patient had acute gastric dilation and was given gastrointestinal decompression. One patient had abdominal pain, which was likely caused by bile entering the pelvic cavity. The patient improved after symptomatic supportive treatment and traditional Chinese medicine physical therapy. Two patients had complications of biliary pancreatitis.

**Conclusion:**

Laparoscopy using a 9Fr disposable pancreaticobiliary catheter could be useful diagnosing and treating common bile duct microstones. These are preliminary results from a descriptive case series.

## Introduction

The annual global incidence of acute pancreatitis is approximately 4.9–73.4 per 100,000 people, with recurrent acute pancreatitis accounting for approximately 21% of affected patients (1, 2). Acute biliary pancreatitis (ABP) is a type of acute pancreatitis caused by biliary system disorders, accounting for 30%–70% of all acute pancreatitis cases. Notably, approximately 20%–40% of ABP cases progress to a severe form of the disease (3). The primary causes of ABP include bile duct stones, roundworm infestation, foreign bodies, endoscopic retrograde cholangiopancreatography, infections, biliary stricture, congenital common bile duct cysts, abnormal congenital pancreaticobiliary junctions, pancreaticobiliary tumors, and sphincter of Oddi dysfunction. Among these, bile duct stones are the most common cause of ABP (4–6).

Common bile duct stones can be classified as primary and secondary common bile duct stones according to their origin. Primary stones form within the bile duct, whereas secondary bile duct stones originate in the gallbladder and migrate into the bile duct. Therefore, accurate diagnosis, appropriate timing of intervention, and selection of surgery are particularly important in the management of ABP. Moreover, microstones in the common bile duct, usually measuring less than 5 mm in diameter, may temporarily block the common channel and even pass into the duodenal papilla. This transient blockage often leads to temporary increases in the levels of bilirubin, liver enzymes, bile enzymes, or amylase. When common bile duct microstones pass through the sphincter of Oddi, they cause mucosal damage, leading to edema and narrowing of the common openings of the biliary and pancreatic ducts. This obstruction disrupts the flow of pancreatic fluid, further increasing the internal pressure in the pancreatic duct. Consequently, the bile flows into the pancreatic duct, and activated pancreatic enzymes can enter the glandular interstitium, leading to autodigestion and ultimately the development of ABP.

The initiating factors of biliary pancreatitis differ from those of other types of pancreatitis (7, 8). Bile duct diseases lead to the simultaneous obstruction of the biliary and pancreatic ducts. This blockage leads to a lack of trypsin in the duodenum, preventing degradation of cholecystokinin-releasing peptides and resulting in increased secretion of cholecystokinin (9). Cholecystokinin receptors on the pancreatic acini activate the calcium signaling pathway and promote the secretion of pancreatic fluid (10). Simultaneously, pancreatic duct obstruction increases the pressure in the pancreatic duct and pancreatic interstitium, and the infectious bile flowing back into the pancreatic duct activates trypsinogen, disrupting the self-protection mechanisms of the pancreas and initiating pancreatitis. When obstruction and reflux persist, they trigger a systemic inflammatory response mediated by the self-digestion of the pancreas, leading to inflammation that progresses from local to systemic and from mild to severe. This course of events results in severe necrotizing pancreatitis characterized by multiple organ dysfunction syndrome.

Enhanced magnetic resonance cholangiopancreatography (MRCP) exhibits good sensitivity and specificity for diagnosing bile duct obstruction. However, its ability to diagnose common bile duct stones, especially small stones with a diameter of less than 5 mm, decreases with increasing bile duct diameter (11, 12). Specifically, the detection rate of common bile duct stones in patients who are MRCP-negative is 54.1%. Moreover, while some smaller common bile duct stones may pass through the duodenal papilla and self-discharge into the intestine, 6.6% of patients still have occult common bile duct stones (13, 14). Therefore, patients who do not undergo biliary exploration are highly likely to develop residual stone-related symptoms postoperatively, often requiring secondary treatment.

The present study aimed to evaluate the clinical efficacy of performing laparoscopy using a 9 Fr disposable pancreaticobiliary catheter to investigate and treat ABP caused by common bile duct microstones. We summarized and reported the clinical data of patients who underwent this type of surgery.

## Materials and methods

### General information

This retrospective case series included consecutive patients who met the criteria for having primary and secondary common bile duct stones. The study was conducted at the Second People's Hospital of Zhangye City, China, from January 2020 to January 2024. Inclusion criteria were as follows: (1) age between 18 and 70 years who had undergone MRCP examination; (2) common bile duct stone diameter less than 5 mm; (3) gallbladder duct diameter 3 mm or greater and common bile duct diameter 5 mm or greater; and (4) American Society of Anesthesiology score 2 points or less, absence of malignant tumors, and complete clinical data available. Exclusion criteria were as follows: (1) pregnancy; (2) diagnosis of a mental illness; (3) presence of a malignant tumor; or (4) incomplete clinical data. This study was approved by the Ethics Committee of Zhangye Second People's Hospital (approval number A033). Written consent was obtained from all patients.

### Surgical methods

**Figure 1:**
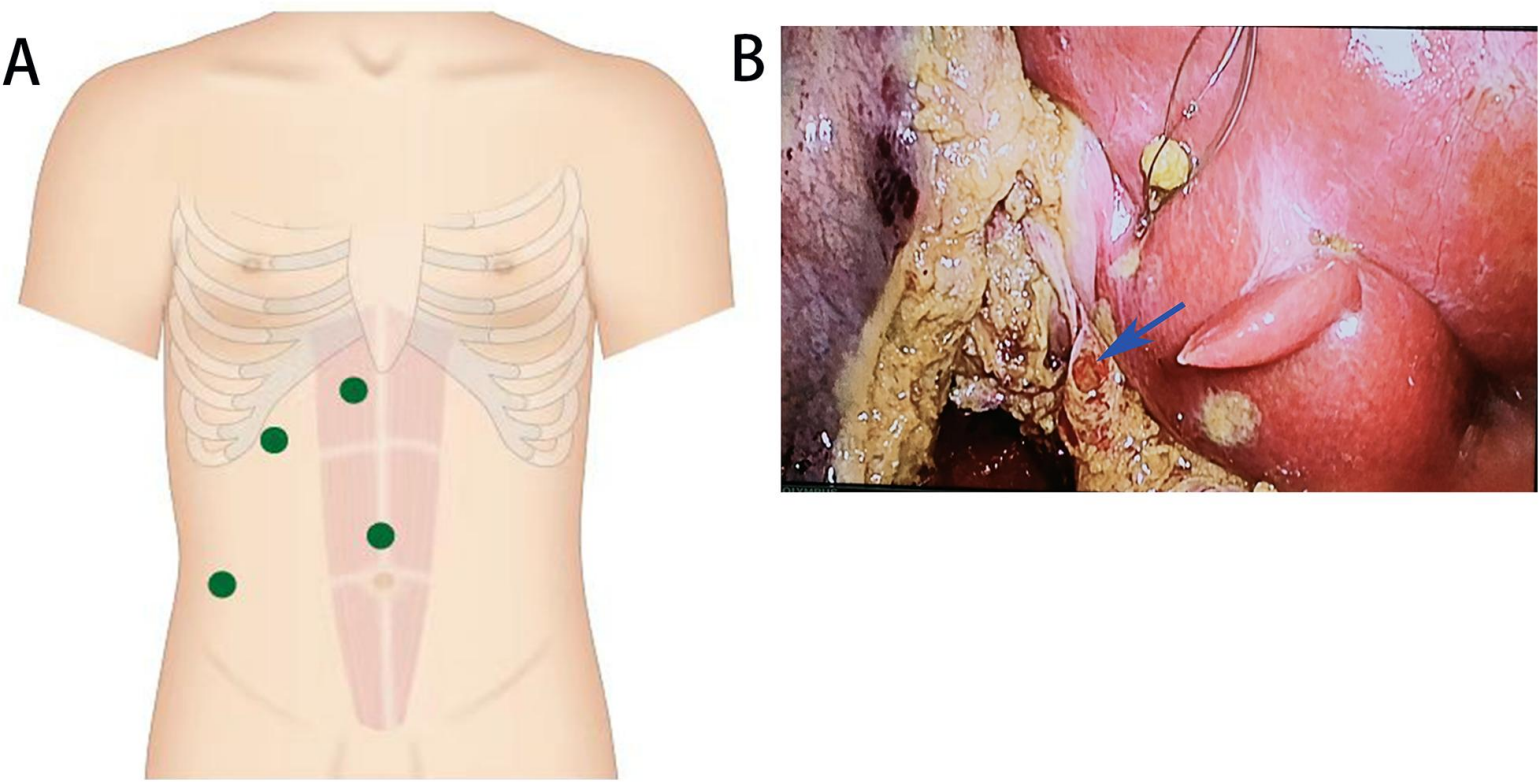
Trocars placed at the umbilical level, above the umbilicus, to the right of the xiphoid process, below the rib margin at the right clavicle midline, and along the anterior axillary line **(A)** A transverse incision (blue arrow) made on the anterior wall of the cystic duct **(B)**.

After initiating general anesthesia, a carbon dioxide pneumoperitoneum was established at a pressure of 12 mmHg. Trocars were placed at the umbilical level, above the umbilicus, to the right of the xiphoid process, beneath the rib margin at the right clavicle midline, and along the anterior axillary line (Figure 1A). The anatomy of the gallbladder triangle, including the cystic artery, cystic duct, and common hepatic duct, was carefully identified and separated. Laparoscopic knotting and suturing techniques were applied: the cystic artery was triple ligated with a 4-0 silk suture, and the cystic duct was also triple ligated with a 4-0 silk suture 1 cm proximal to its junction. A transverse incision was then made on the anterior wall of the cystic duct (Figure 1B).

**Figure 2:**
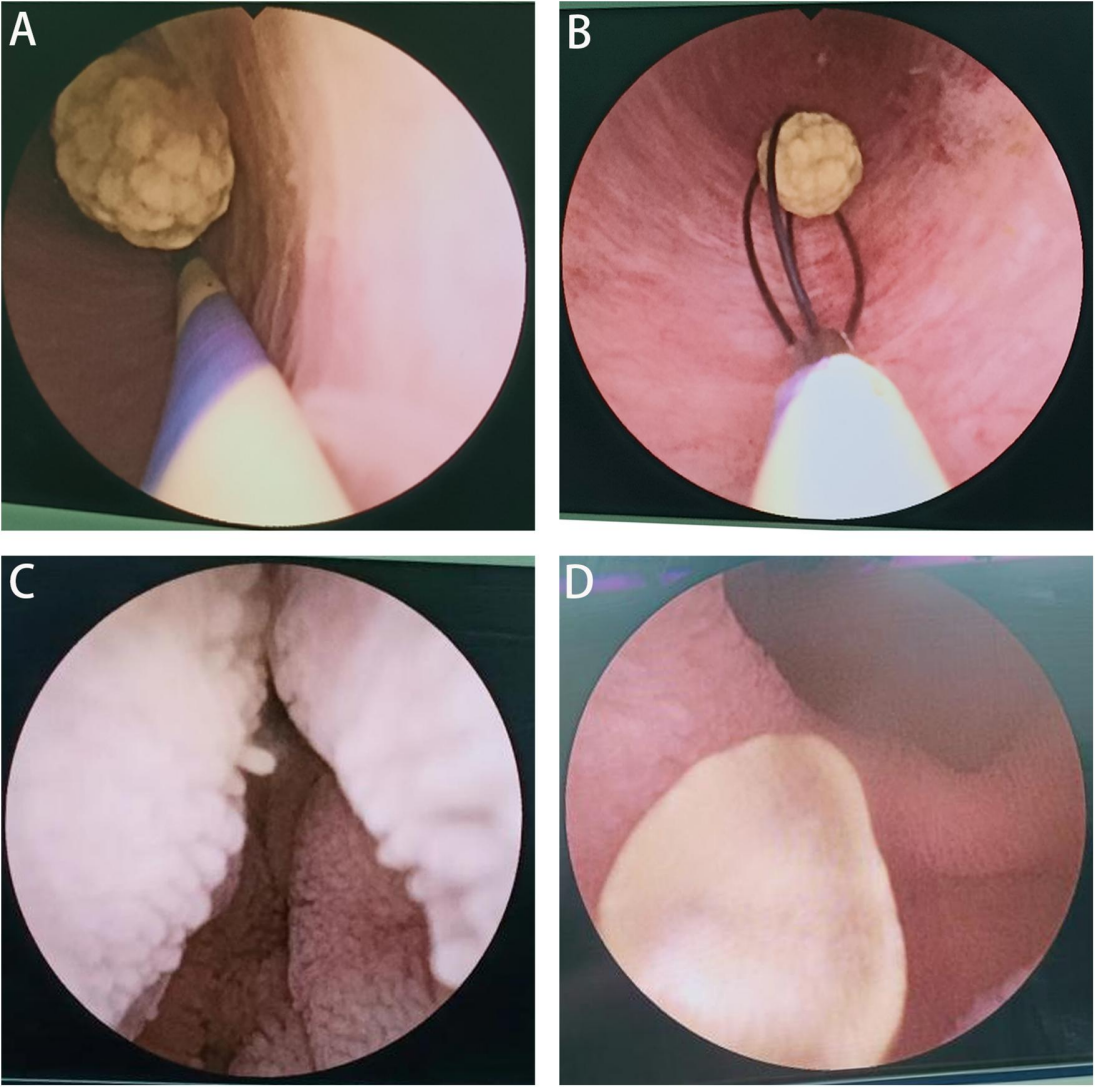
The stones were removed using a disposable pancreaticobiliary imaging catheter combined with a disposable endoscopic spiral stone removal basket **(A)** microstones in the pancreatic common bile duct were removed **(B)** under one-time pancreaticobiliary imaging, the mucosa in the descending duodenum is clearly visible **(C)** microstones in the common bile duct are found in the descending duodenum **(D)**.

After dilation with the tip of a dissecting forceps, under direct vision, the pancreaticobiliary imaging catheter 9 Fr (disposable flexible choledochoscope) was placed in the common bile duct advanced through the ampulla of the hepatopancreas, the large papilla of the duodenum and descending duodenum to the intestinal lumen.for exploration. When stones were found, a disposable endoscopic spiral stone extraction basket was inserted for stone removal (Figure 2A). Microstones located in the pancreatic segment of the common bile duct during exploration were also removed (stone diameter, 3 mm; Figure 2B). After stone removal, the cystic duct was triple ligated with a 4-0 silk suture in preparation for gallbladder removal. If no stones were identified in the common bile duct, it was presumed that the stones were likely small and had temporarily blocked the duodenal papilla. Using the endoscope, the mucosa of the descending duodenal lumen was clearly visualized (Figure 2C), allowing for the removal of any microstones in the common bile duct (Figure 2D). After a thorough examination of the entire pancreaticobiliary system, the gallbladder was removed. At the end of the procedure, an 18 Fr drainage catheter was placed and fixed at the trocar site along the right axillary line near the umbilicus.

### Statistical analysis

Chi-square tests were used to compare categorical variables. Continuous variables are reported using mean ± SD or median (IQR); categorical variables are reported with frequency (%). All analyses were performed using SPSS (version 21.0; IBM Corporation, Armonk, NY, USA). All *P*-values were two-sided, and *P* < 0.05 was considered statistically significant.

## Results

A total of 66 (26 male and 40 female) patients were included. The age of the patients ranged from 23 to 70 years, with a mean age of 51.27 ± 13.95 years. A total of 50 patients had secondary common bile duct stones, and 16 had primary common bile duct stones.

All 66 patients underwent surgery to treat common bile duct stones. The operative time ranged from 80 to 290 min, with a mean of 138.79 ± 36.86 min. Intraoperative blood loss volume ranged from 5 to 50 mL, with mean blood loss of 13.03 ± 7.06 mL. Postoperatively, the mean length of hospital stay was 9.95 ± 2.43 days. We conducted subgroup analyses of patients based on primary and secondary bile duct stones. There were no statistically significant differences in baseline data between the two groups regarding hospital stay; intraoperative bleeding; white blood cell count; and levels of total bilirubin, alkaline phosphatase, amylase, lipase, and gamma-glutamyl transferase preoperatively or postoperatively (Table 1).

**Table 1:** Clinical and pathologic characteristics of patients primary and secondary common bile duct stones.

Characteristics	Total	Primary	Secondary	*P*-value
No. of patients	66	16	50	
Gender				0.444
Male	26 (39.40%)	5	21	
Female	40 (60.60%)	11	29	
Length of hospital stay		8.81 ± 2.23	10.32 ± 2.40	0.015
Intraoperative bleeding		11.56 ± 4.36	13.50 ± 7.71	0.482
Operation time		126.56 ± 21.27	142.70 ± 40.00	0.213
Before surgery WBC		6.82 (5.16–8.82)	6.27 (4.59–9.58)	0.852
After surgery WBC		7.21 (6.20–9.41)	8.23 (6.42–10.45)	0.515
Before surgery TBIL		33.34 (14.55–53.46)	30.58 (15.24–64.92)	0.875
After surgery TBIL		14.67 (11.24–26.58)	19.62 (11.77–36.92)	0.415
Before surgery ALP		166.50 (109.00–248.25)	150.50 (108.75–219.75)	0.670
After surgery ALP		265.00 (105.00–554.25)	127.50 (88.00–175.75)	0.899
Before surgery blood amylase		54.50 (45.00–150.25)	77.50 (57.00–157.50)	0.291
After surgery blood amylase		53.50 (33.00–66.25)	49.50 (41.00–72.00)	0.692
Before surgery rGGT		370.00 (165.25–897.50)	381.50 (162.25–610.50)	0.621
After surgery rGGT		265.00 (105.00–554.25)	262.00 (92.00–408.50)	0.970
Before surgery lipase		48.50 (43.00–66.00)	45.00 (26.75–65.00)	0.274
After surgery lipase		33.50 (21.50–61.00)	45.50 (34.00–75.05)	0.059

WBC, white blood cell count; TBIL, total bilirubin; ALP, alkaline phosphatase; rGGT, Gamma-glutamyl transferase.

The success rate of stone removal was 98.5%. No patients required conversion to choledochotomy or alternative methods. One patient had the stone enter the pancreatic duct during the removal process, resulting in failed stone removal. Postoperative complications occurred in 7 patients. Three patients had fever and improved after antibiotic treatment. One patient had acute gastric dilation and was treated with gastrointestinal decompression. One patient had abdominal pain, which was likely caused by bile entering the pelvic cavity. The patient improved after symptomatic supportive treatment, traditional Chinese medicine, and physical therapy. Two patients had complications of biliary pancreatitis. Due to the common passage, when the choledoscope passes through the large papilla of the duodenum, it stimulates edema of the Oddi sphincter. Postoperatively, the blood amylase and lipase levels increased. After treatment including fasting with water only for 5 days, the patient recovered. All patients were followed for 6–12 months, with no recurrence of common bile duct stones. Follow-up abdominal ultrasonography revealed no abnormalities in the common bile duct or pancreas. Two patients presented with elevated gamma-glutamyl transferase, but no abnormalities were found on biliary ultrasound. They had no other special symptoms and are still undergoing follow-up.

## Discussion

The present study investigated the clinical utility of laparoscopy using a 9 Fr pancreaticobiliary catheter in exploring and diagnosing common bile duct microstones through the duodenum. This strategy utilizes the gallbladder duct approach, which preserves the anatomical integrity of the common bile duct. Notably, bleeding and stenosis of the common bile duct and bile leakage have not been reported with this technique. Moreover, this technique allows for accurate treatment of small stones (<5 mm), especially those located in the liver and pancreatic ampulla, resulting smaller tissue damage and rapid recovery.

Laparoscopic transcystic common bile duct exploration (LTCBDE) has a high success rate in treating patients with choledocholithiasis. However, standard endoscopes or less experienced physicians may have a lower success rate, a higher failure rate for stone removal, and a higher incidence of complications. There are still some difficulties in learning the LTCBDE method. Before learning LTCBDE, it is necessary to accumulate sufficient experience in laparoscopic common bile duct exploration and choledochoscopy. Due to the angle between the cystic duct and the common bile duct, when using choledoscopy to examine the intrahepatic bile duct, the operator needs to have relatively proficient choledoscopy skills to facilitate the operation and avoid residual stones. The slender structure of the 9 F catheter results in a limited working channel diameter. Its removal efficiency for large stones with a diameter >15 mm or multiple complex stones is lower than that of LTCBDE. We chose the pancreaticobiliary imaging catheter (9 Fr) and the disposable endoscopic stone removal basket for biliary microstones. The finer endoscopic operation is gentler and causes less damage, making it a better choice for microstones. Unlike LTCBDE, it can explore the blind area of the liver, pancreas and ampulla and perform operations like stone removal. Use of the 9FR catheter allows adequate judgement whether the relaxation of the duodenal papillary sphincter is good. Combined stone removal with endoscopic retrograde cholangiopancreatography may achieve better results in future clinical research.

The blind areas of the liver, pancreas, and ampulla cannot be clearly visualized using preoperative ultrasound and MRCP. Moreover, most patients cannot undergo 6-mm electronic choledochoscopy intraoperatively. However, both exploration and surgery in the pancreaticobiliary system can be efficiently performed using imaging-guided catheters. A pancreaticobiliary catheter can easily pass through the hepatopancreatic ampulla to explore this region. Smooth passage of the catheter indicates a good level of relaxation in the sphincter of the duodenal papilla (sphincter of Oddi) and suggests that the diameter of the sphincter is at least 3 mm when relaxed. If the procedure, or passage through the organ system, proves challenging, it reflects moderate relaxation of the sphincter of the duodenal papilla, and the diameter of the sphincter is 3 mm or less when relaxed. However, if the catheter cannot pass through the common bile duct but a single-use endoscopic stone basket can, this indicates poor relaxation of the sphincter and that the diameter of the sphincter is at least greater than 1 mm when relaxed. In the present study, the cystic duct diameter needed to be 3 mm or greater and the common bile duct diameter needed to be 5 mm or greater, the above-mentioned limitations are determined by physiological factors. Since the diameter of the 9 Fr choledoscope is approximately 3 mm, if the diameter of the cystic duct is less than 3 mm, the 9 Fr choledoscope cannot pass through the cystic duct. If the diameter of the common bile duct is less than 5 mm, removing the stone basket will be very difficult. Therefore, in appropriate patients, this surgery allows for simultaneous stone removal, integration of diagnosis and treatment, and reduces the risks of residual stones and reoperation. No postoperative bleeding, bile leak, infection or duct injury was found in this study. Pancreatitis occurred in 2 patients after the operation, with an incidence rate of 3%. Our sample size was relatively small, a larger sample will be needed for a controlled study.

In terms of the timing and selection of surgical procedures for ABP, we believe that earlier surgical intervention, ideally within 72 h, is better, provided that blood and urine amylase and lipase levels are only temporarily elevated and enhanced abdominal computed tomography or MRCP reveals no obvious pancreatic exudation. Although gallbladder removal is necessary, it is even more important to examining the common bile duct to fully relieve the obstruction at the common anatomical channel where the gallbladder, pancreas, and intestine meet. This examination should include the duodenal papilla, ampulla of Vater, and the surrounding sphincter complex. Laparoscopy combined with the use of a 9 Fr disposable pancreaticobiliary catheter through the common bile duc is the preferred surgical approach for exploration, as it avoids secondary surgery, shortens hospitalization time, reduces hospitalization costs and patient pain, and ensures patient health.

## Limitations

This study has certain limitations, including its retrospective design, descriptive analysis which lacks a control group, and a relatively small number of patients. The study design is still purely retrospective with no comparator arm; no hypothesis testing, confidence intervals, or subgroup analysis were included; and the findings were reliant on a single-center experience, limiting generalizability. Therefore, this is an exploratory, hypothesis-generating study—not definitive evidence of efficacy.

## Conclusions

This study demonstrates that performing laparoscopy with a 9 Fr disposable pancreaticobiliary imaging catheter to explore the bile duct system may avoid complications such as biliary bleeding and cholangitis associated with T-tube displacement. However, as this was a retrospective, non-controlled, single-center design, future prospective studies with larger sample sizes and robust statistical analyses are needed to validate our findings.

## Data Availability

The original contributions presented in the study are included in the article/Supplementary Material, further inquiries can be directed to the corresponding authors.
