# Correction: Laparoscopic transcystic exploration of the common bile duct using a 9 Fr catheter

**DOI:** 10.3389/fsurg.2026.1818799

**Published:** 2026-03-10

**Authors:** Po Li, Jianrong Cheng, Yiru Hou, Feifei Cui, Xi Chen, Huiling Sun, Ruirui Ma, Jiaxi Yao, Xiaojun Chen

**Affiliations:** 1 Department of General Surgery, Zhangye Second People’s Hospital, Gansu, China; 2 Department of General Surgery, Lanzhou First People’s Hospital, Gansu, China; 3 Department of Medical School, Hexi University School of Medicine, Gansu, China,; 4 Department of Urology, Hexi University Affiliated Zhangye People’s Hospital, Gansu, China; 5 Institute of Urology, Hexi University, Zhangye, Gansu, China

**Keywords:** biliary pancreatitis, choledochoscopy, common bile duct microstones, efficacy, endoscopy

Author Xiaojun Chen was erroneously omitted as corresponding author. The correct corresponding authors are Jiaxi Yao and Xiaojun Chen.

The original version of this article has been updated.

